# Knowledge, Attitude and Acceptability of the Human Papilloma Virus Vaccine and Vaccination Among University Students in Indonesia

**DOI:** 10.3389/fpubh.2021.616456

**Published:** 2021-06-14

**Authors:** Madan Khatiwada, Cissy Kartasasmita, Henny Suzana Mediani, Christine Delprat, Guido Van Hal, Carine Dochez

**Affiliations:** ^1^Network for Education and Support in Immunisation (NESI), Department of Family Medicine and Population Health, University of Antwerp, Antwerp, Belgium; ^2^Faculty of Medicine, Universitas Padjadjaran, Bandung, Indonesia; ^3^Faculty of Nursing, Universitas Padjadjaran, Bandung, Indonesia; ^4^INSERM, U1052, Cancer Research Center of Lyon (CRCL), CNRS UMR_5286, UnivLyon, Université Claude Bernard Lyon 1 (UCBL1), Lyon, France; ^5^Department of Family Medicine and Population Health, University of Antwerp, Antwerp, Belgium

**Keywords:** HPV vaccine, cervical cancer, awareness, vaccine acceptance, University students, Indonesia

## Abstract

**Introduction:** Cervical cancer, a major consequence of persistent HPV infection, is the third most common cancer in women worldwide and has claimed around 311,000 women lives in 2018. The majority of these deaths took place in low- and middle-income countries (LMICs). In LMICs, where cervical cancer screening coverage is low, the HPV vaccine is a promising tool for preventing HPV infections and, thus, averting cervical cancer cases. In Indonesia, cervical cancer is the second most common cancer and HPV vaccination demonstration programs are underway in several provinces, but the HPV vaccine has not yet been introduced nationally. Since students are an important source of information for the community, and medical and nursing students are the future healthcare professionals, this study explored the knowledge, attitude, and acceptability of the HPV vaccine among University students in Indonesia.

**Methodology:** A self-administered online questionnaire was used to assess the knowledge, attitude, and willingness of University students toward HPV vaccination.

**Result:** A total of 433 students from Medical, Nursing, Social Sciences, and other faculties participated in the survey. It was identified that over 90% of the students were aware of cervical cancer and HPV, but only 68% knew about the HPV vaccine before participating in the study. Despite an average knowledge on the HPV vaccine, the students showed a strong willingness to receive the vaccine (95.8% acceptance rate). They believed that the HPV vaccine is safe and effective and that it will protect against HPV infection. The high cost and the lack of adequate information flow on HPV-related topics have been identified as potential barriers to the adoption of the HPV vaccine in Indonesia.

**Conclusion:** Despite a high willingness for HPV vaccine uptake among students, there is a need to provide education on HPV vaccine-related topics to Indonesian students through awareness and training programs and improving the academic curriculum on vaccination for the long-term sustainability of the HPV vaccination program.

## Introduction

Cervical cancer, caused by high-risk genotypes of the Human Papillomavirus (HPV), is the third most common cancer among women worldwide with an estimated 569,847 new cases in 2018, contributing to 6.6% of all female cancers (1). It is the most common HPV-related disease. According to the WHO, in 2018, ~311,000 women died from cervical cancer, 85% of whom occurred in low- and middle-income countries (LMICs) (2). The burden of cervical cancer is extremely high in countries like Indonesia.

Cervical cancer is the second most common cancer in women in Indonesia after breast cancer. Current estimates indicate that each year, 32,469 women are diagnosed with cervical cancer with an incidence rate of 24.5 per 100,000 women and 18,279 die from the disease annually (3, 4). The most prevalent genotypes in Indonesia are HPV16 (35–42%) and HPV18 (28–43%) contributing to around 75–80% of the cervical cancer cases (5).

Cervical cancer can be prevented through regular cervical cancer screening and HPV vaccination (6). However, according to the report from the ICO/IARC HPV Information Center, Indonesia, cervical cancer screening coverage rate was only 24.4% in 2018 (3). Under these circumstances, HPV vaccines are a promising tool for decreasing the incidence and, subsequently, preventing mortality from cervical cancer, as they offer a first level of protection against HPV (7). Currently, three prophylactic vaccines are available against HPV infection. All three vaccines are virus-like particles (VLPs) subunit vaccines. HPV vaccines are highly effective when administered to young adolescents before they become sexually active (8). Studies have shown that HPV vaccines have significantly reduced the HPV infection incidence and the cases of Cervical Intraepithelial Neoplasia 2/3 (CIN2/3) (9).

The HPV vaccine has not yet been introduced in the National Immunization Program (NIP) of Indonesia. However, demonstration programs are underway in five provinces (10). Before the national introduction of the vaccine, it is essential to understand the knowledge, perception, and attitude of the general public regarding cervical cancer, HPV, and HPV vaccination (11). Some studies have pointed out that the public acceptance of the HPV vaccine plays a crucial role to determine the successful introduction of the HPV vaccination program as well as to maintain an optimal vaccination coverage (12–14). In addition, vaccine acceptance reflects the disease risk perception, general vaccination attitude, vaccine safety and efficacy perception, past vaccination history, trust in healthcare workers and the system delivering vaccines, vaccination convenience, and sociodemographic characteristics (15–18). Other studies have also identified knowledge about HPV and HPV vaccines as key predictors for increasing HPV vaccine uptake. Acceptance of the HPV vaccine has increased when parents and young women are well-informed about the risks and benefits of the vaccine (19–21). Despite a low level of knowledge about HPV, HPV vaccine, and cervical cancer, a cross-sectional study on parental acceptance of HPV vaccination in Indonesia showed that the acceptability rate of the HPV vaccine was very high (96.1%) (22). Similar findings were recorded from another study in Yogyakarta province of Indonesia (23).

Our study is the first to be conducted among University students in Indonesia to evaluate the knowledge, perception, and attitude toward HPV vaccine and vaccination. The study in this group is also important because students play a vital role in educating their community about HPV, cervical cancer, and the importance of vaccination. In addition, these students are future parents, and if they are aware of cervical cancer, they can play an important role in making positive decisions related to vaccination for their future children. Moreover, medical and nursing students are future healthcare professionals. Identifying the potential knowledge gaps and barriers to the acceptance of the HPV vaccine among University students can help develop effective educational messages, improve its content to increase the awareness of HPV and HPV vaccine, reduce negative beliefs, and promote HPV vaccine uptake.

## Methods

This study applied a cross-sectional study design with a self-administered online questionnaire to assess the knowledge, attitude, and willingness of University students toward the HPV vaccine and vaccination.

### Questionnaire Development

The questionnaire was developed after an extensive literature review on the determinants of HPV vaccine decision-making, but was not limited to the knowledge, attitude, perception, acceptability, and behavior. The questionnaire was structured into a self-administered anonymous online survey and consisted of 40 items in total. The study was piloted with 12 students from the Nursing Faculty at Universitas Padjadjaran, Bandung, Indonesia to assess the clarity of the questionnaire, and no major modification of the questionnaire was required. The study was conducted in English since the participants in the pilot study highlighted that they were able to understand and interpret the questionnaire.

### Study Design and Participants

The survey was conducted between February 24 and April 23, 2019 on University students enrolled in undergraduate or postgraduate courses at different faculties like Nursing, Medicine, Social science, Communication, Animal husbandry, Fisheries, and Marine sciences at Universitas Padjadjaran, Bandung, Indonesia. The questionnaire was entered into the SurveyMonkey Platform, and the link for the questionnaire was distributed through Gmail and WhatsApp by each Faculty Dean at Universitas Padjadjaran. Responses were recorded in the password protected SurveyMonkey platform. The minimum sample size was 380 based on the assumption that the acceptability rate was 50% with a 5% margin of error and 95% confidence interval. Participation in the study was voluntary and students could withdraw from the study at any time. Students were not paid for their participation. All participants provided their consent before answering the survey questionnaire.

### Ethical Approval

This study was conducted after the review and approval by the Institutional Review Board of Universitas Padjadjaran, Bandung, Indonesia. (Reference number_ 0719101405).

### Measures

The questionnaire included questions on the sociodemographic characteristics, knowledge of HPV, cervical cancer, and HPV vaccine, personal experience of HPV vaccination, perceived facilitators, and barriers to vaccination in general and specific to the HPV vaccine and willingness to accept the HPV vaccine.

Awareness about HPV, cervical cancer, and HPV vaccine was assessed with seven items and the responses were recorded on a two- (1-Yes, 2-No) or three-point scale (1-Yes, 2-No, 3-Do not know). In order to find out the willingness to accept the HPV vaccine, four items were used and responses were also recorded on a three-point scale. Questions to assess students' belief and attitudes toward the HPV vaccine and vaccination were grouped into three constructs: opinion on vaccine and vaccination in general (six items), perceived facilitators (six items), and perceived barriers (eight items). Responses to all items were coded on a five-point Likert scale (1-Strongly Agree, 2-Agree, 3-Neither Agree nor disagree, 4-Disagree, 5-Strongly Disagree).

### Data Analysis

The data was analyzed using the Statistical Software SPSS 25.0. Frequencies and percentages were used to describe the sociodemographic characteristics of the participants, knowledge and views on cervical cancer, HPV, and HPV vaccine, and belief and attitude of students toward HPV vaccination. Chi-square test was used to assess the difference between subgroups with analysis conducted separately by age, faculty, and gender. To test the association between the variables, *p* < 0.05 (two-tailed test) was considered statistically significant. Phi and Cramer's value was used to assess the degree of association. Fisher's exact test was used in the analysis of small samples. Multiple logistic regressions were used to assess the factors associated with HPV vaccine acceptance and barriers to HPV vaccination. Univariate logistic regression analysis was performed to evaluate the predictors of willingness to accept HPV vaccination. Variables with a *p* < 0.10 in the univariate analysis were further entered into the multivariate logistic regression model where adjusted odds ratios (AOR) and their corresponding 95% confidence intervals were calculated if possible.

## Results

In total, 433 students from different faculties of Universitas Padjadjaran participated in the study.

### Sociodemographic Information

The sociodemographic profile of the study population is summarized in Table 1.

**Table 1:** Sociodemographic characteristics of the study participants.

**Characteristics**	**Frequency (*n*)**	**Valid %**
Faculty^*^		
Medicine	237	54.9%
Nursing	119	27.5%
Animal husbandry	39	9.0%
Fisheries and marine sciences	16	3.7%
Social sciences	15	3.5%
Communication science	6	1.4%
Level of Education^*^		
Undergraduate	369	85.4%
Postgraduate	63	14.6%
Age^*^		
Under 20	83	19.2%
20–26	340	78.7%
26+	9	2.1%
Gender^*^		
Male	100	23.1%
Female	329	76.2%
Prefer not to say	3	0.7%

* *One participant skipped the sociodemographic section*.

### Knowledge of Cervical Cancer, HPV, and HPV Vaccine

Out of 430 respondents, 418 (97.2%) stated that they knew about cervical cancer. However, that number decreased for knowledge about the HPV vaccine. Of 372 respondents, 256 (68.8%) were aware of HPV vaccines before participating in the survey, while 116 (31.2%) had no information about the HPV vaccine at the start of this survey. Computational analysis showed a significant association between the knowledge about cervical cancer and faculty (*p*-value = 0.001, Table 2). However, the Fisher exact test showed no association between the knowledge of cervical cancer with gender and age. Additionally, out of 372 respondents, 68.8% (256) believed that cervical cancer was caused by HPV and 86.3% (321) agreed that sexual transmission was vital for the spread of HPV infections among individuals.

**Table 2:** Computation of association between different variables.

**Variables**		***p*-value**	**Odds ratio**	**95% confidence interval**	
				**Lower limit**	**Upper limit**
Knowledge	Faculty	0.001^**^	1.39	1.03	2.18
Knowledge	Gender	0.38	0.59	0.17	1.96
Knowledge	Age	0.96	1.03	0.27	3.95
Vaccine uptake	Faculty	0.001^**^	2.28	1.29	4.03
Willingness to receive the HPV vaccine	Recommend the HPV vaccine to others	0.002^**^	0.20	0.07	0.54
Willingness to receive the HPV vaccine	Knowing someone with cervical cancer	0.98	0.97	0.10	9.62
Willingness to receive the HPV vaccine	Risk perception of HPV infection and cervical cancer	0.93	0.90	0.10	8.05
Willingness to receive the HPV vaccine	History of seeking information on the HPV vaccine	0.01^*^	0.16	0.07	0.26

*p-value significant at < 0.05; p-value < 0.05:*

* *; p-value < 0.005:*

** *. Bold values indicate that the association between variables is significant*.

### Optimal Age for Vaccine Administration and Current Vaccine Use

Of 249 respondents, 35% responded that 15–20 years is the appropriate age for HPV vaccine administration, while 26% and 28%, respectively, thought that 9–14 and 21–26 years were the optimal age group to receive the HPV vaccine.

Among 329 female participants, 246 responded whether they had received the HPV vaccine or not. Of 246 female respondents, 216 (87.8%) answered that they had not received any dose of the HPV vaccine to date. Only 30 respondents had received one or more doses of the vaccine: 13 had received all three doses, 6 had received two doses, 5 had received a single dose, and 6 could not remember the vaccine doses administered to them. Chi-square analysis showed no significant difference in the knowledge about cervical cancer between vaccinated and unvaccinated participants (*p*-value = 1.00) (data not shown).

### Religious and Cultural Barriers to the Adoption of the HPV Vaccine in the Society

Out of 240 respondents, almost 50% (117) stated that there are no religious/cultural barriers in their society regarding vaccination, whereas 27% (67) highlighted that there are negative beliefs such as vaccines that contain haram substances (pig contents) and are not natural, while 23% (56) of the students were unaware of these types of barriers in their society.

### Willingness to Be Vaccinated Against HPV, and Seeking Information About the HPV Vaccine

Participants were asked whether they would accept HPV vaccination if they were within the recommended age when the HPV vaccination program was instigated by the government. Of 238 female respondents, 228 (95.8%) stated that they would receive the HPV vaccination and 217 (96% of 226) participants reported that they would recommend and encourage their family members, relatives, and friends to get the HPV vaccine. More than half of the students (59.7% out of 226 respondents) indicated that they already had information about cervical cancer, HPV, and the HPV vaccine.

Willingness to be vaccinated against HPV and to recommend the vaccine to others were found to be significantly associated (*p*-value = 0.002, Table 2). However, willingness to receive the HPV vaccine was not influenced by knowing someone with cervical cancer (*p*-value = 0.98, Table 2). Moreover, there was also no association between the willingness to accept the HPV vaccine and the perceived risk of HPV infection and cervical cancer among the study participants (*p*-value = 0.93, Table 2).

Multiple regression analysis and ANOVA test depicted that the history of seeking information was significantly associated with the willingness to receive the HPV vaccine (*p*-value = 0.01, Table 2). Some of the important reasons highlighted by the participants in favor of or against HPV vaccination are shown in Table 3.

**Table 3:** Most important reasons in favor of or against HPV vaccination.

**Reasons**	**Frequency (*n*)**	**% of cases**
**In favor of HPV vaccination (*****n*** **=** **225)**		
HPV vaccination can prevent HPV infections.	206	91.56%
HPV vaccine is safe and effective.	106	47.11%
I have good knowledge on cervical cancer, HPV, and HPV vaccine.	141	62.67%
Vaccination is a social norm.	55	24.44%
**Against HPV vaccination (*****n*** **=** **3)**		
The vaccine has many side effects and the injection is painful.	2	66.67%
I am too young to get cervical cancer.	1	33.33%
The vaccine will not protect me throughout my life.	1	33.33%
HPV vaccine is expensive.	1	33.33%

*Participants chose multiple answers giving a total response exceeding 225*.

### Attitude Toward the HPV Vaccine and Vaccination in General

**Figure 1:**
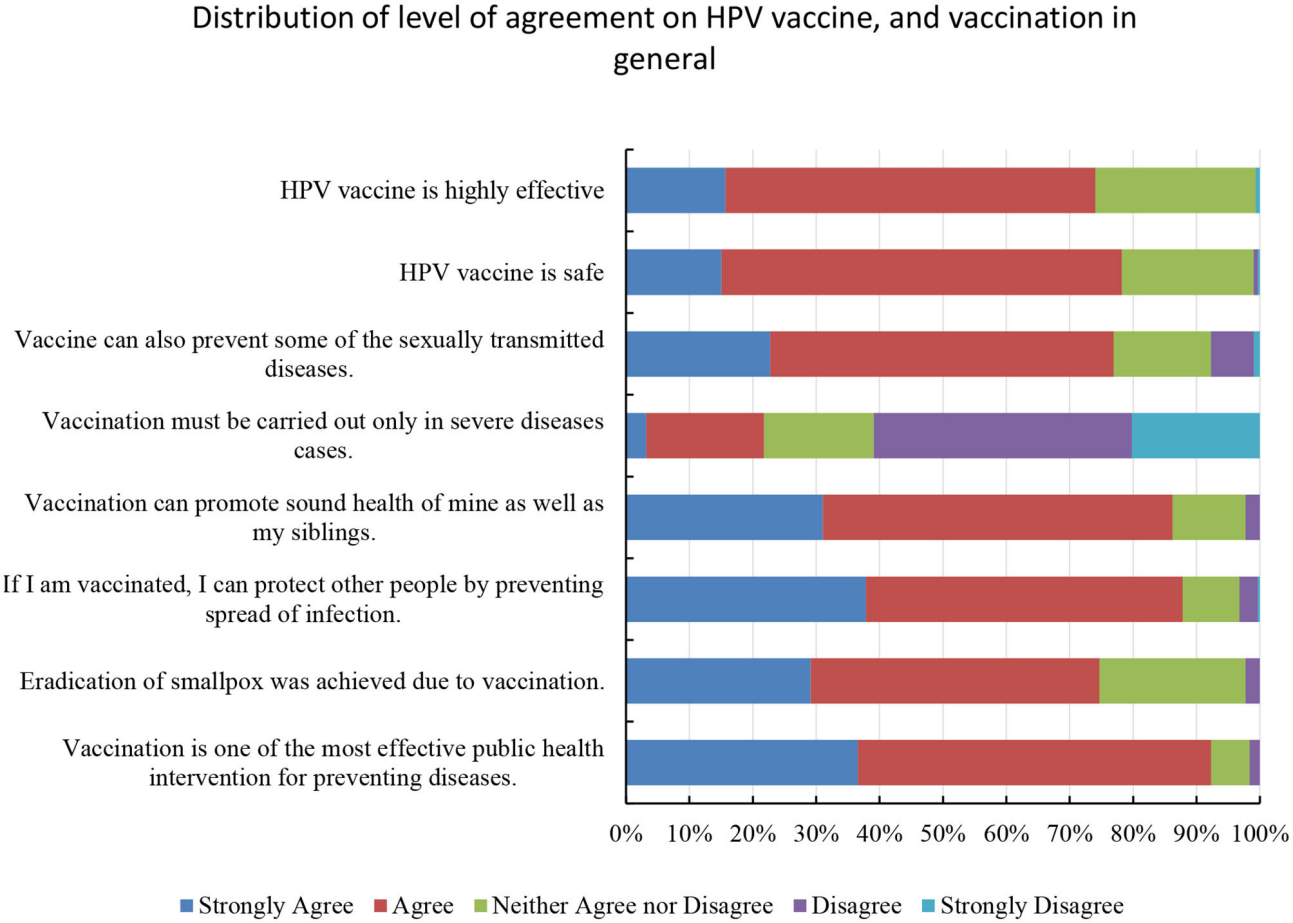
Bar graph illustrating the level of agreement on HPV vaccine and vaccination in general. Participants showed a positive attitude toward the HPV vaccine's safety and efficacy with more than 70% stating that the HPV vaccine is safe and effective. In addition, the participants were also positive toward the vaccine and vaccination in general.

Attitudes toward the HPV vaccine and vaccination in general were determined by asking the participants to give their opinions on eight relevant statements (Figure 1) using the Likert scale. In general, students had positive attitudes toward the HPV vaccine and vaccination in general.

Over 70% of the participants believed that the HPV vaccine is safe and highly effective. However, around 21% of the participants tend to agree that vaccination is not required for healthy individuals.

Of 226 respondents, 143 (63.3%) pointed out that there is a risk of HPV infection even at an early age of 9–14 years old. Of 226 students, 204 (90.3%) stressed that HPV vaccination should be integrated into the NIP in the future.

### Potential Facilitators and Barriers to HPV Vaccination in Indonesia

Potential facilitators and barriers to HPV vaccination in Indonesia were assessed using 14 statements with the Likert-scale. As previously reported, the participants had an overall positive attitude toward the HPV vaccine and believed the HPV vaccine to be safe and effective. Over 60% believed that their families can afford the vaccine and support and encourage them to get HPV vaccination.

In contrast, around 67% of the respondents, out of 312, pointed out that there was not enough information available about cervical cancer, HPV, and HPV vaccine in Indonesia. A quarter of participants also claimed that the HPV vaccine in the private market is expensive and their families cannot afford it.

### Vaccination Venue and Source of Information About the HPV Vaccine

**Figure 2:**
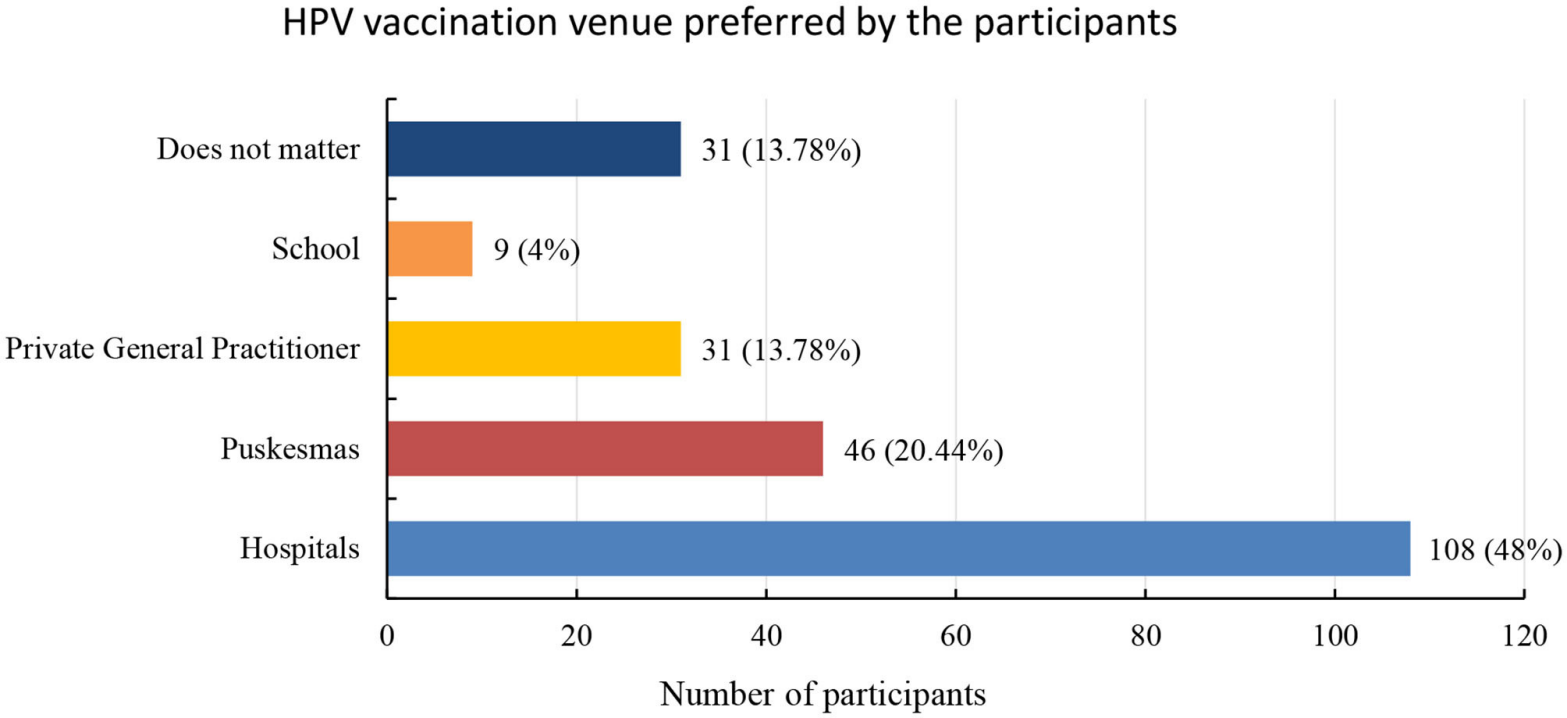
Distribution of HPV vaccination venue preferred by the participants in frequency and valid percentages. Out of 225 respondents, 48% stated that they prefer to go to the hospitals for HPV vaccination followed by Puskesmas (government authorized community health-posts across Indonesia).

Participants were asked where they would prefer to go for HPV vaccination (Figure 2). The majority (48% of 225) stated that they would prefer to go to hospitals for HPV vaccination followed by Puskesmas (20.4%).

**Figure 3:**
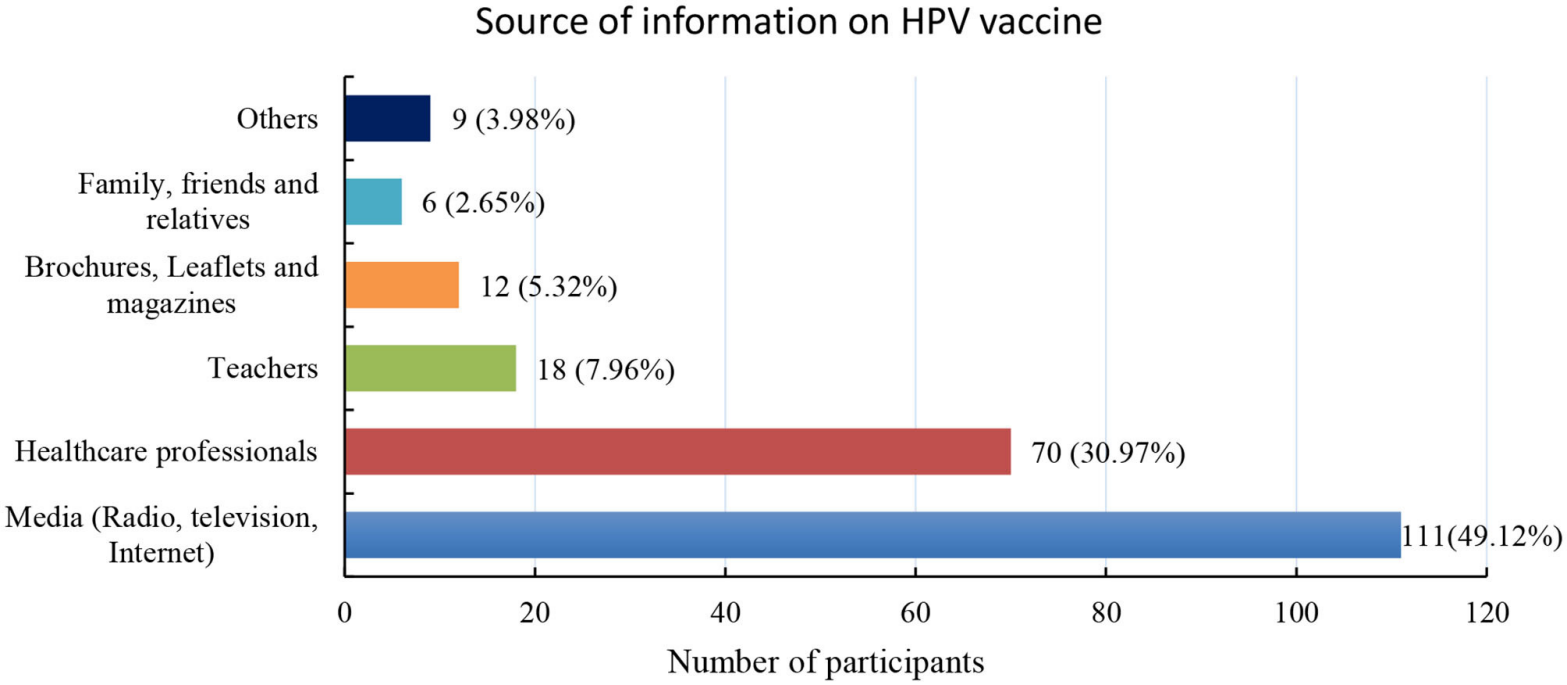
Distribution of source of information the participants obtained on the HPV vaccine in frequency and valid percentages. Media is the most popular source of information on HPV vaccination in Indonesia (49.12%) followed by healthcare professionals (30.97%).

In addition, sources of information about HPV, cervical cancer, and HPV vaccination were also assessed (Figure 3). Media was the main source of information about the HPV vaccine, followed by healthcare professionals.

## Discussion

The main aims of this study were to provide an insight on the level of knowledge about cervical cancer, HPV, and HPV vaccine among University students, as well as to understand their willingness to accept HPV vaccination if introduced into the NIP, and to identify the potential facilitators and barriers which may have an impact on the decision to accept the vaccine.

In this study, 97.2% of the participants knew about cervical cancer, which infers that the majority of the University students are well-equipped with cervical cancer knowledge. Over 90% claimed that HPV is the leading cause of cervical cancer, indicating that the students are knowledgeable about HPV. In contrast, just over two-thirds of the participants had heard of the available HPV vaccines, thus, demonstrating a knowledge gap and communication deficit leading to a low awareness on the HPV vaccine. In a similar study in health sciences students in Malaysia, it was found that 88.2% (241) of students out of 273, believed cervical cancer to be a serious disease, 80.2% (219) pointed out that HPV is the main cause of cervical cancer, and 78.7% (215) conjectured that the HPV vaccine can prevent cervical cancer (24). The main reason for a better knowledge about the HPV vaccine in Malaysia is the fact that the vaccine has been introduced in the NIP of Malaysia since 2010 and the coverage rate until 2017 was 83–91% for the full vaccination course (25). This study also shows that a greater exposure to health information can lead to a better knowledge (25). However, a study conducted at the University of Lagos, Nigeria, among female students reported that out of 368 participants, only 214 (58.1%) were aware of cervical cancer, 64 (17.4%) had knowledge on HPV infection, and 52 (14.1%) knew about the HPV vaccine (26), indicating a low knowledge of HPV infection and existence of the HPV vaccine. These findings also illustrate that people are likely to become more aware of the HPV vaccine once it is introduced in the NIP.

A previous study among Indonesian parents showed that only 16.6% knew about HPV, <16% had heard of HPV vaccination, and about 40% had no knowledge about HPV, HPV vaccination, and cervical cancer (22). Collectively, these data indicate that the level of knowledge about HPV-related topics was low among Indonesian parents compared to University students, but this study on Indonesian parents was conducted a couple of years ago. Our recent study showed that the University students have an adequate knowledge on cervical cancer and HPV but were less informed about the available HPV vaccines. In order to increase the knowledge about HPV vaccines, it is important to develop and disseminate educational messages about the HPV vaccine and organize vaccine awareness programs at the University and school levels along with awareness programs to reach drop-out students in the community. In addition, the academic curriculum could be improved to include HPV and cervical cancer preventive measures. Furthermore, students have an adequate understanding of HPV transmission since over 85% pointed out that HPV is transmitted sexually in correlation with the findings among University students in Italy where 464 (89.8%) out of 517 participants stated that HPV is a Sexually Transmitted Infection (27).

More than a third of the participants thought that 15–20 years old is the appropriate age for HPV vaccine administration and about 28% believed that 21–26 years old is the optimal age group for HPV vaccination, which assumes that in Indonesia, youngsters start sexual activity in the later parts of their life and probably after marriage and are also less aware of the recommended age for HPV vaccination. Only 26% indicated 9–14 years old as the appropriate age for HPV vaccination, as per the recommendation of the WHO (28). Similarly, a study conducted on Turkish Nursing students revealed that 363 (69.8%) out of 520 students knew that the HPV vaccine is recommended for females aged 9–26 years (29).

The HPV vaccine in Indonesia is only available in the private market, resulting in a high cost and, probably, beyond the affordability of most of the middle-income families. This was reflected in the data because only 30 (12.2%) of the total 246 respondents had received the HPV vaccine before the onset of the study. This was similar in a study in Lebanon on female college students, where 16.7% out of 215 participants had been vaccinated with the HPV vaccine before starting the survey (30). In contrast, 40.5% of the students had received at least one dose of HPV vaccination before getting enrolled in the study in Italy (27). The vaccine uptake rate in Indonesia is to be expected to substantially increase after providing the vaccine for free through the NIP, as many students have described the cost of the vaccine as one of the main obstacles to the adoption of the HPV vaccine.

Despite an average knowledge of the HPV vaccine, the vaccine acceptance rate among Indonesian University students is immensely high at 95.8%, which is much higher than the acceptance rate in Nigeria (26) and Turkey (29) where only 57.7% and 66.3% of the students, respectively, were willing to receive the HPV vaccine. A study conducted in Mozambican adolescent girls in 2017 showed that 91% (1,025 out of 1,130 participants) were willing to be vaccinated if HPV vaccination was available in Mozambique (31). In Malaysia, the vaccine acceptance rate was as high as 83.8% (24). The willingness to receive HPV vaccination varies in different regions of the world. However, although the level of knowledge about the HPV vaccine was average among the students in our study, they are well-informed about cervical cancer and HPV, which might have resulted in the high acceptance rate of the HPV vaccine. Unlike the study in Malaysia (24), in our study, willingness to accept the HPV vaccine was not associated with HPV risk perception. This may be because the study participants consider themselves to be at a low risk of developing cervical cancer from HPV infection because they are still young.

In addition, about 96% of the students tend to recommend the HPV vaccine to their family members and friends who are within the recommended age for vaccination. This is higher than in Lebanon (30) where 72% would recommend the vaccine to their college friends. The main reason for the strong recommendation as well as the willingness to get HPV vaccination is that the majority of the students believed that HPV infection can be prevented by vaccination and that the HPV vaccine is safe and effective.

Overall, students have a positive attitude toward HPV vaccination. More than 90% of the students emphasized that the HPV vaccine should be provided as part of the NIP, making it accessible to a large number of people. The inclusion of HPV vaccination in the NIP has become a huge success in countries like Australia where cervical cancer is expected to be eliminated by 2035 (32).

The major potential facilitators highlighted by the students were that HPV vaccines are safe and effective and can protect against HPV infection. However, a study in Turkey showed that 55.6% of the students were unsure of the vaccine effectiveness and 28.9% were afraid of the side effects of the vaccine (29). Thus, these findings suggest that Indonesian students have more positive attitudes toward the HPV vaccine and vaccination than Turkish students.

Moreover, a majority of the students pointed out that a lack of sufficient information about cervical cancer and HPV vaccine in Indonesia and the high price of the vaccine were major barriers to HPV vaccination. These findings correlate with the previous study in Indonesian parents, where it was also shown that insufficient information about HPV, cervical cancer, and HPV vaccines and high cost of the vaccine, are two major barriers to HPV vaccine uptake (22). Thus, a better organized and sustainable awareness program and national policies to fully or partially subsidize the HPV vaccination program must be implemented.

The preferred choice to receive HPV vaccination was the hospital followed by Puskesmas, while the school was the least desired location. Since this study was conducted in Bandung city, which is the fourth biggest city in Indonesia, the participants find it more convenient to go to a hospital for vaccination. However, Puskesmas was chosen as the second option because many people in Indonesia belong to middle-income families and Puskesmas are cheaper than hospitals.

Almost half of the students stated that media (radio, television, and internet) were the main source of information on HPV vaccination in Indonesia, followed by healthcare professionals and teachers. Similar results were obtained in Nigeria (26) and Turkey (29), where participants described media and internet as the main source of HPV information in their countries, respectively. Therefore, these media can be used effectively to disseminate credible information about HPV, cervical cancer, and HPV vaccine in Indonesia. Moreover, healthcare professionals and teachers can also be used to provide information about the HPV vaccine, since they are highly trusted by the general public and students, respectively.

The study had some limitations as it was limited to one University in Indonesia. Therefore, it is difficult to generalize the findings. In addition, the study was conducted online through WhatsApp and Gmail, which are expected to be commonly used in this population but a few students with a lower socioeconomic status may have missed out. The attempt to minimize non-random selection bias was made by sending survey reminders to faculties that had a minimal response. Despite these reminders, more responses were received from the medical faculties (Medical and Nursing), which could have created a selection bias.

## Conclusion

To sum up, University students have a good knowledge of cervical cancer and HPV but are slightly behind in having up-to-date information regarding the HPV vaccine. Despite a slightly insufficient knowledge about this vaccine, the students showed a strong willingness to receive the HPV vaccine and encourage others to get vaccinated. Students, in general, have a positive attitude toward the HPV vaccine and vaccination, but highlighted insufficient circulation of information about cervical cancer, HPV, and HPV vaccine in their community. They also identified the high cost of the vaccine to be one of the main barriers to HPV vaccine uptake. Furthermore, the majority of participants believed that the HPV vaccine prevents lifelong HPV infection and that the vaccines are safe and effective. Based on our study, it is recommended that the media can be widely mobilized to raise awareness about HPV vaccination in Indonesia, along with the active engagement of healthcare professionals. It is also equally important to involve religious leaders, ministry of health representatives, health organization representatives, and healthcare professionals in the organization of vaccination awareness programs to ensure that the correct message will be perceived by the general public.

## Data Availability Statement

The raw data supporting the conclusions of this article will be made available by the authors, without undue reservation.

## Ethics Statement

This study was conducted after review and approval by the Institutional Review Board of Universitas Padjadjaran, Bandung Indonesia. (Reference number_ 0719101405).

## Author Contributions

CDo and CK developed the original idea and are the supervisors of the study. Co-supervisors of the study are HM, CDe, and GV. MK conducted the study as part of his master studies developing the protocol and questionnaire, analyzing data and report writing, under supervision of all co-authors. CK and HM assisted with the ethical clearance and data collection. All authors reviewed the data analysis, report writing, gave input into the manuscript, and are equally responsible for the content of the manuscript.

## Conflict of Interest

The authors declare that the research was conducted in the absence of any commercial or financial relationships that could be construed as a potential conflict of interest.
